# Expenditure on Paid-for Gambling Advertising During the National COVID-19 ‘Lockdowns’: An Observational Study of Media Monitoring Data from the United Kingdom

**DOI:** 10.1007/s10899-022-10153-3

**Published:** 2022-08-29

**Authors:** Nathan Critchlow, Kate Hunt, Heather Wardle, Martine Stead

**Affiliations:** 1 Institute for Social Marketing and Health, University of Stirling, FK9 4LA Stirling, Scotland; 2 School of Social and Political Sciences, University of Glasgow, Glasgow, Scotland

**Keywords:** Gambling, Advertising, Marketing, COVID-19, Lockdowns, Pandemic

## Abstract

Changes in gambling advertising during national COVID-19 ‘lockdowns’, when stay-at-home rules restricted participation in certain gambling activities, provides important context to variance in gambling behaviour during these periods. This study describes expenditure on paid-for gambling advertising during three national lockdowns, compares expenditure to pre-pandemic estimates, and compares changes in expenditure by subsector. Data come from an observational study of weekly expenditure on paid-for gambling advertising in the United Kingdom (*n* = 135 weeks; beginning 2019 to mid-2021), focusing on three COVID-19 lockdowns: (1) March-May 2020; (2) November-December 2020; and (3) January-March 2021. We descriptively analysed how total advertising expenditure in each lockdown (£GBP, inflation-adjusted) compared to the same time points in 2019, both overall and by subsector (bookmakers, lotteries, online bingo, online casino and poker, gaming, pools, mobile content). Gambling advertising expenditure during lockdown one was 38.5% lower than 2019 (£43.5 million[m] vs. £70.7 m, respectively), with decreases across all subsectors (range: -81.7% [bookmakers] to -2.8% [online bingo]). Total advertising expenditure in lockdown two was 49.3% higher than 2019 (£51.7 m vs. £34.6 m), with increases for 5/7 subsectors (range: -31.6% [mobile content] to + 103.8% [bookmakers]). In lockdown three, advertising expenditure was 5.9% higher than 2019 (£91.2 m vs. £86.1 m), with increases for 4/7 subsectors (range: -92.4% [pools] to + 49.2% [mobile content]). Reductions in advertising expenditure in lockdown one are congruent with self-reported reductions in overall gambling also observed during this period. Further research is needed to determine whether increased advertising expenditure in lockdowns two and three correlates with increased gambling, overall and for specific subsectors.

## Introduction

The COVID-19 pandemic brought unprecedented disruption to daily life in 2020 and 2021. In the United Kingdom (UK), as elsewhere, this included periods of national ‘lockdown’ to suppress virus transmission, when all non-essential premises were closed and ‘stay-at-home’ rules were mandated for all but those in necessary roles (Brown & Kirk-Wade, 2021; Institute for Government, 2021). These lockdowns had a significant impact on the gambling industry, with land-based gambling premises forced to close (e.g., betting shops, casinos, bingo halls) and, during the initial lockdown in 2020, the cancellation of most professional live sport (British Broadcasting Corporation [BBC], 2020).

Multiple studies have reported overall reductions in gambling during the initial lockdowns, both in the UK and elsewhere (Brodeur et al., 2021; Clarke et al., 2021; Hodgins & Stevens 2021). Overall reductions, however, mask important trends, such as variations among gambling subgroups and engagement with individual gambling activities. During the first lockdown in the UK, for example, a cross-sectional survey of regular sports bettors reported that approximately one-in-six had started at least one new gambling activity and one-in-three had increased frequency on at least one activity (Wardle et al., 2021). Moreover, a cross-sectional survey of adults reported limited change in frequency for the most-involved gamblers (Sharman et al., 2021) and a longitudinal survey of young adults reported increases in online gambling (e.g., online poker or casino) in both regular and occasional gamblers (Emond et al., 2022). A review of this emerging evidence suggests that consistent correlates of increased gambling during the lockdowns included severity of problem gambling, being of younger age, and being male (Hodgins & Stevens, 2021).

To date, most self-report research in the UK has focused on the first national lockdown. There is limited comparable data for the second and third lockdowns, which were implemented November to December 2020 and January to March 2021, respectively. These later lockdowns remain important to consider given that many professional live sporting events had resumed by (and continued during) these lockdowns and companies had had further time to adapt their business models and product offerings to the pandemic market conditions, thus altering the parameters compared to the initial lockdown. Industry monitoring data also highlights the importance of examining longer-term trends during the pandemic. For example, in the 2020/2021 financial year, which included lockdowns two and three and most of lockdown one, there were increases in gross gambling yield for the remote casino, betting, and bingo subsectors compared to the previous year (Gambling Commission, 2021a). Increased uptake of internet (online/remote) gambling is important given that such formats (e.g., continuous play) are a risk factor for problem gambling (Allami et al., 2021).

Enforced changes in the availability of some gambling activities, for example through the closure of land-based premises, is undoubtedly a key determinant of consumer gambling patterns during the lockdowns. Nevertheless, it is also important to examine market forces which may have had a concurrent influence on these trends, both overall and for different gambling subsectors. One such factor is advertising, which research has consistently reported to be associated with gambling outcomes (Beynon et al., 2021; Gunter 2019; Newall et al., 2019). Investigating gambling advertising during the lockdowns is particularly important in the context of problem gambling, as those experiencing increased severity of gambling problems are reportedly more susceptible to the influence of advertising (Binde & Romild, 2019) and problem gambling has been identified as a factor associated with increased gambling during lockdowns (Hodgins & Stevens, 2021).

There is a growing body of literature documenting the extent and nature of gambling marketing in the UK (e.g., Ginnis & Kitson, 2019; Kilick & Griffiths 2022; Sharman et al., 2022; Torrance et al., 2021) and reach among consumers (e.g., Djohari et al., 2019; Ginnis & Kitson, 2020; Torrance et al., 2020). These observations, however, may not generalise to during the COVID-19 pandemic, particularly national lockdowns, when mitigation measures reduced opportunities for engagement with some advertising and gambling activities. To date, there is little publicly available data concerning gambling advertising activity during the pandemic. There are survey data on self-reported awareness of gambling advertising across three time points in 2020 – which show that six-in-ten recall seeing gambling adverts or sponsorships at least once a week – but these findings are not isolated to lockdown periods (Gambling Commission, 2021b). This means there is also limited research examining the impacts of voluntary industry initiatives to minimise harm during lockdowns, for example the decision by a representative trade body in the UK to voluntarily remove all television and radio advertising for gaming products between the end of April and start of June 2020 (i.e., during the first lockdown) (Betting and Gaming Council, 2020a).

This study responds to the lack of contextual information on gambling advertising during the COVID-19 pandemic through analysis of data collected by a leading media monitoring company. Specifically, we: (1) describe expenditure on paid-for gambling advertising during the three national COVID-19 lockdowns in the UK; (2) compare advertising expenditure to pre-pandemic estimates; and (3) compare changes in advertising expenditure by gambling subsector (e.g., bookmakers, lotteries, online casino and poker etc.). This study also briefly reports trends in gambling advertising expenditure outwith the lockdowns. This builds on similar pre-pandemic estimates (Ginnis & Kitson, 2019) and provides context to on-going gambling policy debates in the UK, such as the Gambling Act review (Department for Digital, Culture, Media, and Sport [DCMS], 2020).

## Methods

### Design

We conducted an observational study of weekly expenditure on paid-for gambling advertising in the UK using data purchased under licence from Nielsen’s Advertising Intelligence Services. The study formed part of a larger project, the ‘Betting and Gaming COVID-19 Impact Study’, which was established to examine how the pandemic had influenced gambling trends and harms in the UK (Hunt et al., 2020). Expenditure data is a recognised method for examining advertising activity over a given period and Nielsen data have been previously used to examine advertising expenditure trends for gambling, alcohol, and e-cigarettes (Ginnis & Kitson, 2019; Jernigan & Ross 2020; Stead et al., 2021; White et al., 2015).

### Observation Periods

Data were provided in weekly intervals, from week commencing 31st December 2018 to week commencing 26th July 2021 (*n* = 135 weeks). Weekly intervals provided greater sensitivity to detect changes during lockdowns versus monthly or quarterly intervals. Within the time series, three periods were identified for national COVID-19 lockdowns in the UK, broadly defined as when stay-at-home rules were mandated with limited exceptions. These periods were defined using government reporting of restrictions, mostly in relation to England (Brown & Kirk-Wade, 2021: Institute for Government, 2021). As data were in weekly intervals they do not always coincide with the exact dates on which lockdowns commenced or concluded, but they are aligned as closely as possible.

Lockdown one was defined as week beginning 23rd March 2020 (date the first lockdown began) to week ending 31st May 2020 (restrictions on leaving home were lifted in England 1st June). During lockdown one, most live professional sport was suspended in the UK and elsewhere (BBC, 2020). Lockdown two was defined as week beginning 2nd November 2020 (second lockdown began in England 5th November) to week ending 6th December 2020 (lockdown lifted 2nd December). Lockdown three was defined as week beginning 4th January 2021 (third lockdown began in England 6th January) to week ending 28th March 2021 (stay-at-home order lifted 29th March). Most live sports had resumed by lockdown two and continued thereafter, mostly with no spectators (or limited numbers) permitted. Detailed information on restrictions across the UK, how they were phased out towards the end of each lockdown, and variations across the devolved nations are reported elsewhere (Brown & Kirk-Wade, 2021; Scottish Parliament Information Centre, 2022; Senedd Research, 2022).

### Advertising Activities and Gambling Subsectors Observed

The data purchased under licence from Nielsen covered nine paid-for advertising activities: cinema, television, direct mail (i.e., mailshots to named addressee), door drops (i.e., bulk mailshots without addressee), internet (e.g., web banners), outdoor (e.g., billboards), press (e.g., newspapers), radio, and digital video. Nielsen estimate expenditure for each advertising activity, including relevant discounts for bulk purchases, using their complex proprietary media monitoring methodology. Sources of expenditure data vary by media type, but include market rate cards which detail the cost of advertising space, data supplied direct from clients, and data supplied by advertising brokers/agencies. Expenditure data for most activities were recorded at least weekly, except for direct mail, door drop, and cinema advertising, which were recorded monthly. Nielsen divided monthly estimates for these activities across the weeks in each month to estimate weekly expenditure, although we note that this may not always directly reflect when and how the advertising activity took place. The data were segmented at three levels: operator, subsector, and advertising (media) activity. For this analysis, expenditure data are reported both overall and for the seven gambling subsectors indexed in the Nielsen database: bookmakers, gaming, lotteries, online bingo, online casino and poker, pools, and mobile content (i.e., promoting smartphone applications). All expenditure data were reported in Pound Sterling (£GBP).

### Analysis

Data were descriptively analysed and visualised using a combination of SPSS (version 27) and Microsoft Excel. For each week, we first calculated total expenditure on paid-for gambling advertising and the number of operators with any recorded expenditure in that week, both overall and separately for each subsector. We then adjusted the nominal weekly expenditure estimates to 2021 prices using the Consumer Prices Index including owner occupiers’ Housing costs (CPIH), the lead inflation measure for the UK (Office for National Statistics, 2017). To perform this adjustment, each year was divided into four 13-week blocks and adjusted in relation to the relevant quarterly CPIH Index; Q4 2020 had an additional week as 2020 was a leap year. For reference, the CPIH increased 5% between Q1 2019 and Q3 2021 (index range: 106.7 to 112.0; Base year = 2015).

We then descriptively analysed how expenditure on paid-for gambling advertising during each lockdown compared to the equivalent period (i.e., the same weeks) in 2019. This provided a means of comparing lockdown trends to those pre-pandemic while minimising any confounding influence from seasonality. For lockdown three, which occurred between January and March 2021, data were still compared against 2019 to avoid any confounding influence from the early stages of the pandemic in 2020; for example, some sporting events had been postponed in Europe a month before the first lockdown began in the UK (Guardian, 2020). For expenditure, we examined how total inflation-adjusted expenditure across each lockdown compared to 2019. For operators, we examined how the average number with any spend in each week over the lockdowns compared to 2019. We examined both nominal and relative (%) changes, the latter of which provided an opportunity to descriptively compare trends within and between lockdowns. All changes were computed both overall and separately for each gambling subsector. All analyses were computed on the full expenditure values, but these are rounded to the nearest hundred thousand in the results (e.g., £2,275,000=£2.3 m [million]).

## Results

### Overview of Gambling Advertising Activity Across the Observation Period

**Fig. 1:**
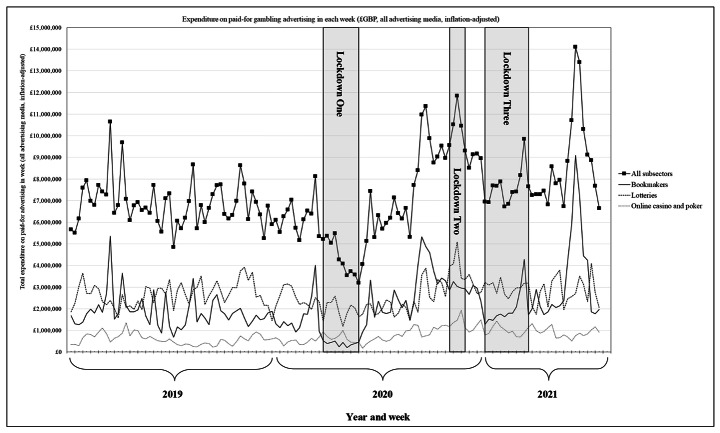
Total expenditure on paid-for gambling advertising in each week (£GBP, inflation-adjusted) for all subsectors and separately for the bookmaker, online casino and poker, and gaming subsectors. **Notes**: Subsectors shown are illustrative of one which appeared heavily affected by COVID-19 lockdowns (bookmakers, sport cancelled in lockdown one and with limited/no supporters in lockdowns two and three) and two subsectors which appeared less affected (online casino and poker and lotteries); Expenditure is after discounts applied by Nielsen and adjusted to Q3 2021 prices using the CPIH

**Fig. 2:**
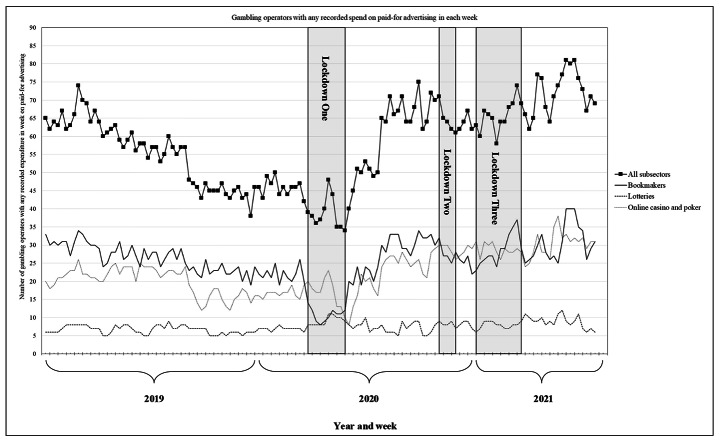
Number of gambling operators with any recorded spend in each week for all subsectors combined and separately for the bookmaker, online casino and poker, and lottery subsectors. **Notes**: Subsectors shown are illustrative of one which appeared to be heavily affected by COVID-19 lockdowns (bookmakers, sport cancelled in lockdown one and with limited/no supporters in lockdowns two and three) and two subsectors which appeared less affected (online casino and poker and lotteries)

Across the 135 weeks, an estimated £972.5m was spent on paid-for gambling advertising across all subsectors and media (adjusting for inflation). In total, 233 operators were recorded as having some advertising expenditure in at least one week. Advertising expenditure in 2019 was an estimated £356.2m (*M*=£6.8m per week) with 131 operators having some recorded spend (*M* = 55.7 operators per week; *SD* = 8.7). Advertising expenditure in 2020 was an estimated £368.3 m (*M*=£6.9m per week) with 148 operators having some recorded spend (*M* = 53.7 operators per week; *SD* = 12.0). Over the 30 weeks observed in 2021, advertising expenditure was an estimated £248.0m (*M*=£8.3m per week) with 149 operators having some recorded spend (*M* = 69.5 operators per week; *SD* = 6.2). Weekly time series of advertising expenditure and the number of operators with any expenditure in each week are reported in Figs. 1 and 2, respectively.

### Gambling Advertising During Lockdown One

During lockdown one (10 weeks, March to May 2020), inflation-adjusted expenditure on paid-for gambling advertising was 38.5% lower than the equivalent period in 2019 (£43.5 m vs. £70.7 m, respectively) (Table 1) (Fig. 1). There were decreases in advertising expenditure for all subsectors, with the largest relative decrease for bookmakers (-81.7%; £3.9 m vs. £21.5 m) and the smallest for online bingo (-2.8%; £8.4 m vs. £8.7 m). During lockdown one, the average number of gambling operators with any recorded advertising expenditure in each week was also 37% lower than the equivalent period in 2019 (38.6 vs. 61.3 operators) (Fig. 2). This decrease was observed across all subsectors, except lotteries, which increased 33.8% (9.1 vs. 6.8 operators).

**Table 1:** Total expenditure on paid-for gambling advertising during the three lockdowns (£GBP, inflation-adjusted), the average number of operators with any recorded advertising expenditure in each week during the three lockdowns, and comparison to the equivalent weeks in 2019 (overall and by subsector)

Lockdown	Gambling subsector							
	**Overall**	**Bookmakers**	**Gaming**	**Lotteries**	**Online bingo**	**Online casino and poker**	**Pools**	**Mobile content**
**One (Weeks 13–22; March to May 2020)**								
Total spend (£GBP, millions)	43.5	3.9	2.8	19.2	8.4	6.6	1.4	1.1
Change vs. same period in 2019 (%)	−38.5	−81.7	−47.7	−16.4	−2.8	−21.2	−42.7	−17.1
Average *n* operators with any spend in each week (*SD*)	38.6 (*4.4*)	10.8 (*1.8*)	4.2 (*1.9*)	9.1 (*1.3*)	8.5 (*2.1*)	17.1 (*4.0*)	1.0 (*0.0*)	1.5 (*1.4*)
Change vs. same period in 2019 (%)	−37.0	−61.2	−46.8	+ 33.8	−22.0	−24.3	−60.0	−66.7
**Two (Weeks 45–49; November to December 2020)**								
Total spend (£GBP, millions)	51.7	15.0	2.6	19.9	6.2	7.1	0.6	0.3
Change vs. same period in 2019 (%)	+ 49.3	+ 103.8	+ 70.8	+ 23.7	+ 29.8	+ 93.0	−25.5	−31.6
Average *n* operators with any spend in each week (*SD*)	64.6 (*3.9*)	27.8 (*2.6*)	9.2 (*1.3*)	8.2 (*0.8*)	9.4 (*1.1*)	28.8 (*1.8*)	3.0 (*0.0*)	5 (*0.7*)
Change vs. same period in 2019 (%)	+ 46.2	+ 25.2	+ 91.7	+ 46.4	+ 27.0	+ 94.6	+ 87.5	+ 66.7
**Three (Weeks 1–12; January to March 2021)**								
Total spend (£GBP, millions)	91.2	24.0	6.4	35.9	11.6	11.8	0.1	1.3
Change vs. same period in 2019 (%)	+ 5.9	−3.2	+ 2.5	+ 15.5	−12.9	+ 48.0	−92.4	+ 49.2
Average *n* operators with any spend in each week (*SD*)	65.6 *(4.3)*	28.7 (*4.4*)	8.3 (*0.9*)	7.9 (*1.0*)	9.8 (*1.6*)	28.8 (*1.8*)	2.0 (*0.0*)	6.1 (*1.2*)
Change vs. same period in 2019 (%)	−0.3	−7.5	+ 22.2	+ 10.5	−26.4	+ 33.7	−27.3	+ 62.2

**Notes**: Expenditure is after discounts applied by Nielsen and adjusted to Q3 2021 prices using the CPIH; All spend values reported to the nearest hundred thousand.

### Gambling Advertising During Lockdown Two

During lockdown two (five weeks, November to December 2020), inflation-adjusted expenditure on paid-for gambling advertising was 49.3% higher than the equivalent period in 2019 (£51.7 m vs. £34.6 m) (Table 1) (Fig. 1). Compared to 2019, there were increases in advertising expenditure for most subsectors, particularly bookmakers (+ 103.8%; £15.0 m vs. £7.4 m), online casino and poker (+ 93.0%; £7.1 m vs. £3.7 m), and gaming (+ 70.8%; £2.6 m vs. £1.6 m). There were decreases in advertising expenditure for pools (-25.5%; £0.6 m vs. £0.8 m) and mobile content (-31.6%; £0.3 m vs. £0.4 m). During lockdown two, the average number of gambling operators with any recorded advertising expenditure in each week was also 46.2% higher than the equivalent period in 2019 (64.6 vs. 44.2 operators) (Fig. 2). This increase applied across subsectors, ranging from bookmakers (+ 25.2%, 27.8 vs. 22.2 operators) to online casino and poker (+ 94.6%, 28.8 vs. 14.8 operators).

### Gambling Advertising During Lockdown Three

During lockdown three (12 weeks, January to March 2021), inflation-adjusted expenditure on paid-for gambling advertising was 5.9% higher than the equivalent period in 2019 (£91.2 m vs. £86.1 m) (Table 1) (Fig. 1). Compared to 2019, advertising expenditure was higher for online casino and poker (+ 48.0%; £11.8 m vs. £8.0 m), mobile content (+ 49.2%; £1.3 m vs. £0.9 m), lotteries (+ 15.5%; £35.9 m vs. £31.1 m), and gaming (+ 2.5%; £6.4 m vs. £6.2 m). There were decreases in advertising expenditure for pools (-92.4%; £0.1 m vs. £1.8 m), online bingo (-12.9%; £11.6 m vs. £13.4 m), and bookmakers (-3.2%; £24.0 m vs. £24.8 m). During lockdown three, there was little change in the average number of gambling operators with any recorded advertising expenditure in each week compared to the equivalent period in 2019 (-0.3%; 65.6 vs. 65.8 operators) (Fig. 2). There was a decrease in the average number of operators with any recorded advertising expenditure for pools (-27.3%; 2.0 vs. 2.8 operators), online bingo (-26.4%; 9.8 vs. 13.3 operators), and bookmakers (-7.5%; 28.7 vs. 31.0 operators). There were increases for mobile content (+ 62.2%; 6.1 vs. 3.8 operators), online casino and poker (+ 33.7%; 28.8 vs. 21.5 operators), gaming (+ 22.2%, 8.3 vs. 6.8 operators), and lotteries (+ 10.5%; 7.9 vs. 7.2 operators).

## Discussion

We used media monitoring data to observe expenditure on paid-for gambling advertising across the three national COVID-19 lockdowns in the UK and compared expenditure to the same time points in 2019 (i.e., pre-pandemic). To our knowledge, this is the first study to examine trends in gambling advertising during the pandemic. Consistent with wider advertising trends in the UK (Sweney, 2020), inflation-adjusted expenditure on gambling advertising during the initial lockdown (March to May 2020) was almost two-fifths lower than in 2019, with decreases across all subsectors. By contrast, advertising expenditure in lockdown two (November to December 2020) was almost 50% higher than during the equivalent period in 2019, with increases for most subsectors. Lockdown three (January to March 2021) only had a modest increase in advertising expenditure versus 2019, which is largely consistent with pre-pandemic trends (Ginnis & Kitson, 2019). Across the full years of data available, expenditure on paid-for gambling advertising was similar in 2019 and 2020, despite the significant pandemic disruption in 2020.

For the online casino and poker and gaming subsectors, there were increases in both total advertising expenditure and the average number of operators with any weekly spend during lockdown two and three, compared to 2019. These advertising trends are congruent with the increased gross gambling yield also reported for the remote casino subsector during the 2020/2021 financial year, which included almost all of the three lockdowns observed (Gambling Commission, 2021a). Taken together, these increases may plausibly suggest that lockdowns created favourable conditions to defend or expand market share for some remote gambling products and online operators. Increased expenditure on lottery advertising in lockdowns two and three (vs. 2019) is also consistent with the increased gross gambling yield reported for the National Lottery in the 2020/2021 financial year, which industry discourse suggests was also partly attributable to increased focus on remote sales (Neville, 2021).

Expenditure on bookmaker advertising during lockdown one (March to May 2020) was around four-fifths lower than in 2019. This reduction in advertising expenditure coincided with the cancellation of most live sport during the initial lockdown, which reduced both the breadth and volume of gambling products and markets to promote (BBC, 2020). Expenditure on bookmaker advertising during lockdown two (November to December 2020), however, was approximately double that of 2019. This may reflect increased intensity of advertising to capitalise on the resumption of sport and efforts to compensate for revenue lost during the first lockdown. There is also evidence of capitalising on the resumption of sport outwith the lockdowns, with increases in expenditure on bookmaker advertising (relative to 2019) also occurring: (1) when the Premier League returned in June 2020, following its suspension during the initial lockdown (Premier League, 2020a); (2) at the start of start of the new football season in September 2020 (Premier League, 2020b; English Football League, 2020); and (3) during the delayed 2020 European Football Championships, which instead took place summer of 2021 (Union of European Football Associations, 2020). Expenditure on bookmaker advertising in lockdown three (January to March 2021) was slightly lower versus the same period in 2019. Further investigation is required to determine whether this represents a genuine reduction in advertising expenditure or displacement to other marketing activities not captured in the Nielsen data (e.g., social media and sponsorship).

Decreased expenditure on paid-for gambling advertising during the first lockdown is consistent with the overall reductions in gambling also reported by consumers in the UK during this period (e.g., Sharman et al., 2021; Wardle et al., 2021). However, given the reported links between marketing and gambling, it is now also important to consider whether increased advertising expenditure during lockdowns two and three (November to December 2020 and January to March 2021, respectively) was reflected in increased gambling among consumers, both overall and for specific subgroups and activities. It is particularly important to examine whether increased advertising expenditure for some remote (online) gambling subsectors during lockdowns two and three was reflected in increased uptake of such activities, and sustained engagement over time, given that they are a known risk factor for problem gambling (Allami et al., 2021) and those experiencing problem gambling are reportedly more susceptible to the influence of advertising (Binde & Romild, 2019). Although the opportunity to gather self-report data like those extensively reported for the first lockdown (Hodgins & Stevens, 2021) has passed, changes could still be examined using industry data covering the later lockdowns.

Decreases in total advertising expenditure and the average number of operators with any weekly spend during lockdown one is also consistent with a voluntary industry initiative to remove all television and radio advertising for gaming products, announced partway through the initial lockdown (Betting and Gaming Council, 2020a). The same representative body reaffirmed their commitment towards a series of measures to protect staff and consumers at the start of lockdown two, including a pledge to ensure ‘appropriate and responsible advertising including monitoring volume’ (Betting and Gaming Council, 2020b; Betting and Gaming Council, 2020c). It is unclear, however, whether the direct commitment to voluntarily stop or reduce advertising was repeated in lockdowns two and three. Increased advertising expenditure in both lockdowns (vs. 2019) would suggest not, or least only limited reductions. If there were no further reductions, the rationale for why this step was considered necessary initially, but not subsequently, remains unclear. That the commitment in lockdown one also only related to some advertising activities, some gambling products, and some operators, also highlights the challenges of ensuring consistency and maximising the effectiveness of harm-reduction approaches using voluntary mechanisms. This contrasts with Spain, for example, where statutory restrictions were reportedly placed on most gambling advertising activities to limit harm during the pandemic (iGamingBusiness, 2020).

The data have several practical applications. First, they provide important context to the reductions in gambling reported by consumers during the initial lockdown. At least in the case of advertising, the data also show that the market conditions in subsequent lockdowns were not the same as the first. This highlights an important need to further examine consumer trends across the pandemic, particularly if those experiencing problem gambling were more susceptible to increased advertising in later lockdowns (Binde & Romild, 2019). The data also update existing estimates of expenditure on paid-for gambling advertising in the UK (Ginnis & Kitson, 2019), taking into account the impact of the pandemic. This provides context to concurrent policy debates, for example the UK Government’s review of its Gambling Act, which is considering controls on advertising (DCMS, 2020). The data also show the utility of advertising expenditure data to examine trends in the UK gambling market. Future research could use such data in econometric models to examine whether trends in advertising – overall and for specific subsectors – are predictive of short, medium, and longer-term trends in gambling and harms. Possible outcome data include gross gambling yield, self-reported gambling patterns and harms, or industry data. Econometric studies do have limitations (Binde, 2014; Saffer, 2020), but there is little research of this nature for gambling advertising and alcohol industry documents have shown that such methods are used to judge the impact of marketing campaigns (Maani-Hessari et al., 2019).

Some limitations to our analysis should be acknowledged. Expenditure only provides a proxy for advertising volume and intensity and does not account for other factors that mediate or moderate the influence of advertising (e.g., design features, offers, or inducements). Expenditure was estimated by Nielsen using an extensive range of data sources, but some margin of error is inevitable and it is likely that not all advertising activity is captured. While the data cover a wide range of paid-for advertising media, they are not exhaustive of all the activities used to promote gambling in the UK, such as social media and affiliate marketing (Houghton et al., 2019), platform marketing (Stead et al., 2016), or sponsorship (Purves et al., 2020). We only compare lockdown trends to one equivalent time-point pre-COVID-19 and cannot directly show how the data relate to longer-term trends or to a year with a different pattern of sporting events (e.g., high profile international tournaments such as World Cups). A longer time series would have also provided the ability to predict expenditure in 2020 and 2021, assuming no pandemic disruption, which could have acted as a counterfactual to the trends observed. We also only focus on gambling advertising; data were not available to directly compare to broader advertising trends in the UK, including variations in the cost of advertising media across the pandemic. Finally, the data are only descriptive of advertising activity and cannot determine motive or impact, for example defending or growing market share versus increasing uptake or frequency of gambling. Nevertheless, the investment observed in this study suggests that advertising must make some positive contribution to the economic model which underpins the gambling industry in the UK.

## Conclusions

Compared to the same periods in 2019, inflation-adjusted expenditure on paid-for gambling advertising decreased during the first national COVID-19 lockdown in the UK, but increased during lockdowns two and three, substantially so in lockdown two. Decreased advertising expenditure in lockdown one correlates with reductions in overall gambling also reported by consumers during this period. It is therefore important that future research considers to what extent, if at all, increased advertising expenditure in lockdowns two and three was mirrored by increased gambling, both overall and for specific subsectors and subgroups (e.g., those experiencing gambling problems). The data also provide updated estimates concerning advertising expenditure, which may help inform ongoing policy debates about the nature and control of gambling marketing in the UK, such as the review of the Gambling Act.

## Data Availability

The data analysed during this current study are not publicly available under the licence agreement between the researchers and Nielsen’s Advertising Intelligence Services. Reasonable requests to access the data can be made to the authors, but these require review and permission from Nielsen. Data can otherwise be purchased from Nielsen.
